# Multiple pathways to scaling up and sustainability: an exploration of digital health solutions in South Africa

**DOI:** 10.1186/s12992-021-00716-1

**Published:** 2021-07-06

**Authors:** Alison Swartz, Amnesty E. LeFevre, Shehani Perera, Mary V. Kinney, Asha S. George

**Affiliations:** 1 Division of Social and Behavioural Sciences, School of Public Health and Family Medicine, University of Cape Town, Cape Town, South Africa; 2 Department of Epidemiology, School of Public Health, Brown University, Providence, USA; 3 Department of International Health, Johns Hopkins Bloomberg School of Public Health, Baltimore, USA; 4 Division of Public Health Medicine, School of Public Health and Family Medicine, University of Cape Town, Cape Town, South Africa; 5 School of Public Health, University of the Western Cape, Cape Town, South Africa

**Keywords:** Digital health solutions, Scale up, Sustainability, South Africa, Health systems

## Abstract

**Background:**

With the aim to support further understanding of scaling up and sustaining digital health, we explore digital health solutions that have or are anticipated to reach national scale in South Africa: the Perinatal Problem Identification Programme (PPIP) and Child Healthcare Problem Identification Programme (Child PIP) (mortality audit reporting and visualisation tools), MomConnect (a direct to consumer maternal messaging and feedback service) and CommCare (a community health worker data capture and decision-support application).

**Results:**

A framework integrating complexity and scaling up processes was used to conceptually orient the study. Findings are presented by case in four domains: value proposition, actors, technology and organisational context. The scale and use of PPIP and Child PIP were driven by ‘champions’; clinicians who developed technically simple tools to digitise clinical audit data. Top-down political will at the national level drove the scaling of MomConnect, supported by ongoing financial and technical support from donors and technical partners. Donor preferences played a significant role in the selection of CommCare as the platform to digitise community health worker service information, with a focus on HIV and TB. A key driver of scale across cases is leadership that recognises and advocates for the value of the digital health solution. The technology need not be complex but must navigate the complexity of operating within an overburdened and fragmented South African health system. Inadequate and unsustained investment from donors and government, particularly in human resource capacity and robust monitioring and evaluation, continue to threaten the sustainability of digital health solutions.

**Conclusions:**

There is no single pathway to achieving scale up or sustainability, and there will be successes and challenges regardless of the configuration of the domains of value proposition, technology, actors and organisational context. While scaling and sustaining digital solutions has its technological challenges, perhaps more complex are the idiosyncratic factors and nature of the relationships between actors involved. Scaling up and sustaining digital solutions need to account for the interplay of the various technical and social dimensions involved in supporting digital solutions to succeed, particularly in health systems that are themselves social and political dynamic systems.

## Introduction

In sub-Saharan Africa, the number and scope of digital solutions used in health is rapidly increasing. Despite this rapid proliferation, few digital solutions reach national or semi-national scale, and even fewer are used on a sustained basis. Several factors lend themselves to the development, national deployment and effective use of digital health solutions at scale [1]. In South Africa, these include a policy environment that supports innovation with oversight over the delivery of digital programmes and services, skilled and experienced technical partners, high levels of mobile phone penetration, gender parity in phone access, moderate to high levels of digital literacy, and the availablity of resources necessary to support digital solutions' development and use [2–6].

Despite such advantages, very little attention has been paid to understanding the processes that underpin efforts to scale up and sustain digital health solutions in South Africa or elsewhere [7, 8]. We understand “scale” as moving beyond small, time-bound pilot projects, to increased geographic coverage and more widespread use. Many definitions of sustainability exist with most encompassing continued programme activities, with fewer definitions including continued health benefits, capacity built, ongoing adaptation and ability to recover costs [9]. In this article, we define sustainability as the ability of a digital solution for health to “[persist] long term” [10]. Emerging literature points to the complex and multi-dimensional nature of the process of scaling digital solutions for health [10, 11]. Multiple domains require consideration, including the condition the solution is trying to address, the technology and its perceived value, as well as the adopters, organisational context and wider environment surrounding it. The broader social, political and historical contexts where tools are implemented also play a significant role in the ways that solutions are embedded and adapted over time, and potentially sustained within a health system [12, 13].

With the aim to further assist health policy and systems learning on scaling up and sustaining digital health, we examine factors underpinning the scale and potential sustainability of digital health solutions in South Africa. We focus on four varied cases of digital health solutions that have reached, or are anticipated to reach, national scale in South Africa. These include the Perinatal Problem Identification Programme (PPIP), Child Healthcare Problem Identification Programme (Child PIP) that are mortality audit reporting and visualisation tools, MomConnect, a direct to consumer maternal messaging and feedback service and CommCare, a community health worker (CHW) data capture and decision-support application. Based on these cases we explore key elements that are particular to scaling up and potentially sustaining digital solutions in this context, that could be more broadly applicable to other low and middle income countries.

## Methods

This study used a qualitative case study design [14]. Cases were selected based on 1) implementation at current or proposed national scale; 2) variation in the current stage of scaling; and 3) recommendations from key stakeholders in the National Department of Health (NDOH). For each case, key informants were purposively sampled for maximum variation to include: government employees holding a range of positions, technical partners, non-governmental organisations (NGOs), donors and academics. In addition to the initial purposive sample of participants, additional participants were identified using a snowball sampling technique, until saturation was reached.

Our final sample included 32 participants: NDOH policy makers (*n* = 3), government advisors (*n* = 5), government implementers (*n* = 6), non-government organisations (NGOs; *n* = 2), technology companies (*n* = 9), academics (*n* = 4), and donors (*n* = 3). As our primary aim was to understand the initial decision-making processes behind how and why particular solutions scaled, end-users were not included in our participant sample. Semi-structured interviews were conducted based on an interview guide that drew from the study’s conceptual framework. Interviews took place either in person or via Skype and lasted approximately 1.5 hours. Interviews were transcribed and coded using Dedoose, a web 2.0 application that supports the analysis of qualitative research data.

**Fig. 1:**
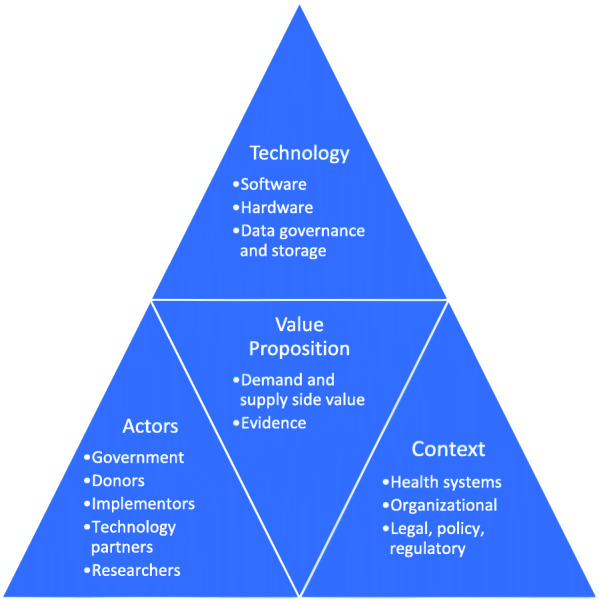
Conceptualizing the factors that drive the scale up of digital health solutions (developed by authors informed by [10, 16–18])

We developed a framework adapted from Greenhalgh’s non-adoption, abandonment, scale-up, spread, and sustainability (NASSS) framework of technology scale-up, supported by further insights from the wider scaling up literature [15–17]. The initial codebook was informed by this literature and refined iteratively in conversation with the research team. Further thematic analysis was conducted on the drivers of and barriers to scaling and potential sustainability. These include: value proposition, actors, digital health solution and implementation strategy, and context (Fig. 1). Under ‘value proposition’ we explore characteristics of each solution that were valued by respondents, including the evidence that informed use and decisions to scale. The section on ‘actors’ explores the multiple motivations, experiences and interactions between a wide range of actors involved in or affected by these digital solutions. In the theme on ‘technology’ we explore the technical aspects that are relevant to scaling the digital health solution, including software (intellectual property and licensing), hardware and data governance and storage. The final section on ‘context’ looks at the broader health systems context including health workers use and response; organisational structures including national committees, task teams; and legal, regulatory, and policy environment.

Verbal informed consent was obtained from all participants. Ethical clearance was obtained from the University of Cape Town, South Africa (HREC Ref: 121/2019). Quotes were designated to respondents in the following categories: 100 s- government, 200 s- donor, 300 s-CommCare, 400 s- MomConnect, 500 s- PPIP and Child PIP and 600 s- academics who could speak to the digital health landscape in South Africa. Some respondents spoke to more than one case study. Draft versions of the manuscript were shared and discussed with key policy makers and researchers working in South Africa before the article was finalised.

## Results

Table 1 in the Appendix descriptively introduces the four South African cases explored. Cases are presented in order of initial implementation (oldest to most recent), beginning with PPIP and Child PIP, followed by MomConnect that was implemented in its current form at national scale in 2014. At the time of interviews, the CommCare application was selected for implementation at a national scale to support the work of CHWs who form part of the ward-based primary healthcare outreach teams (WBPHCOTs) in over 20 President’s Emergency Plan for AIDS Relief (PEPFAR) priority districts. However, it has yet to scale across the country.

In the thematic sections that follow, findings highlight the enabling and challenging factors related to value proposition, actors, technology and organisational context of each digital solution. PPIP and Child PIP are presented together due to the similar nature of these solutions.

### PPIP and Child PIP

#### Value proposition

PPIP and Child PIP were established to assist in improving quality of care and strengthening the South African public health system through supporting and improving the process of mortality audits, data management and analysis. The electronic input of data is integral to this process because it enables improved data access, synthesis and visualisation. These features are reported to be particularly helpful for facility level managers and staff: “it’s a fantastic tool for managers because being a digital system it can actually do all that print work for you. You don’t have to spend hours drawing up graphs” (502_Govt. advisor).

Respondents spoke to the benefit of PPIP and Child PIP in simplifying the process of mortality reviews by saving time and making the data more readily accessible and intelligible to a wider audience who could see “the value and the power of having collated data” (501_Govt. policymaker). Data were often used in mortality audit meetings, with printed graphs displayed publicly in some facilities (502_Govt. advisor).

Programme data analysed and visualised by these solutions were seen as critical to supporting providers’ advocacy for improvements in care. As one early PPIP advocate put it: “that was for me the value of PPIP. It was really in the hands of the clinician and it gave the clinician that evidence to negotiate for better services” (402_Govt.advisor). One early Child PIP advocate described the programme as fulfilling a “personal need” to improve care in the facilities (504_Govt. implementor). Others spoke to the value of data highlighting potential gaps in health worker training, as well as to substantiate requests to higher levels of the health system for additional equipment to support clinical practice on the ground. In this sense, access to this digitised data enabled clinician-led advocacy across health system levels.

However, increased access to digital data also heightened the perceived threat of punitive action at provincial, facility and individual levels. As an academic put it: “Data [are] used often for performance measurement and then you know you have this carrot/stick thing where *oh now you’ve met this quota, now you need to do more* or - you know” (601_ academic). In the early days of PPIP, data was analysed by the South African Medical Research Council (SAMRC) and University of Pretoria Maternal and Infant Health Strategies Unit and shared with the NDOH and other stakeholders. Knowledge that the data was shared may have contributed to the perception that it was being used 'against' poorly performing provinces. Although these solutions are not intended to justify blame for avoidable deaths at individual or facility levels, this was occasionally expressed as a concern (402_Govt.advisor).

#### Actors

A core set of PPIP and Child PIP champions, predominantly obstetrician gynaecologists, neonatologists and midwives for PPIP and facility-based paediatricians for Child PIP, developed and drove uptake in facilities across the country, along with its mainstreaming into routine service delivery. Leadership for PIPP was provided by a senior obstetrician and gynaecologist who holds a range of national and international appointments, serves as an advisor to World Health Organization (WHO) Geneva, and has had his work recognised as a model for replication elsewhere in the region. He was able to marshal resources, authority and convening power to drive programme activities, including annual mortality meetings. These meetings provided implementers with a chance to share their experience, showcase data and learnings and foster an important set of broader social connections. For Child PIP, the initial group of advocates were described as being “like a family”, with an “organically” developed oversight team of clinicians in five provinces, North West, Mpumalanga, Western Cape, Free State and the Eastern Cape (504_ Govt. implementer).

Neither technology partners nor external donors played a significant role in scaling PPIP and Child PIP. At various points, small amounts of funding supported circumscribed activities, including initial programme development and annual mortality meetings. Technology partners have recently played an important role in updating and revitalizing programme software but unlike the initial and sustained engagement with MomConnect and CommCare, this involvement is limited to a one-off activity.

#### Technology

PPIP and Child PIP are relatively simple software programmes that support data capture, analysis, and data visualisation. PPIP software was designed in Clarion in 1995, but is currently written in Windows. In 2019, in response to maintenance challenges and frustrations expressed by users that it was slow and outdated, efforts were undertaken to upgrade the software for Child PIP. The upgraded Child PIP is a web application that works off-line, allowing for future connectivity in the event that reliable internet access becomes more widely available in health facilities. As one respondent explained: “As part of upgrade efforts, technologies (React frontend, .NET core API, and Electron shell) have been used which allow Child PIP to be installed as a desktop application on any operating system (Windows, Mac, Linux). This will additionally allow the application to eventually be run as a website with minimal changes to the code” (405_tech. company). While plans are underway to upgrade the software for PPIP, at the time of interviews updates had not been made.

One of the challenges underpinning PPIP and Child PIP applications is they have to be downloaded onto a computer because there remains limited internet connectivity in health facilities where data are collected and recorded (405_tech. company). The process of obtaining permissions to download the software was noted as an obstacle to timely use. A government implementer of Child PIP noted that it could take six to nine months “to go through [this] whole bureaucratic rigmarole” (506_Govt. implementor). In cases where facilities did not have the resources to upgrade computers or hardware, or replace what may have been damaged or stolen, clinicians were allowed to use their personal computers. Despite possible issues of data security, respondents reported that all of the information was secure and confidential (i.e. without naming individual patients or staff). This was critical to the success of the programme because in discussions about avoidable deaths, when  “everything’s confidential then you’re more likely to get to the truth than people making up stories to protect themselves” (503_ Govt. tech. assistance).

Once inputted, PPIP and Child PIP data are typically housed on facility (or provider) computers, with centrally-based servers in Pretoria for PPIP. While cloud-based storage was explored, the cost was deemed prohibitive; as a one respondent put it “a hell of a cost” (503_Govt. tech. assistance). Lastly, neither PPIP or Child PIP are interoperable with other government health information systems at a national level, including District Health Information Software version 2 (DHIS2). While the reasons for this are largely procedural – i.e. lack of commitment and supporting resources – the end result is that of a dual parallel mortality reporting system (502_ Govt. advisor).

#### Organisational context

PPIP and Child PIP were initially developed and implemented more than 20 years ago, which has allowed these solutions to become more embedded in the health system, with national-level engagement evolving over time. Some respondents expressed frustration at the initial lack of senior National Department of Health leadership behind PPIP and Child PIP (506_ Govt. implementor; 502_ Govt. advisor; 101_Govt. NDOH. policy). When compared to MomConnect, PPIP and Child PIP were described by one respondent as “not sexy”, referring to the lack of widespread enthusiasm, including from external funders, about these solutions (101_ Govt. NDOH. policy).

Respondents explained that support for PPIP and Child PIP was driven from the ground up, but at a national level, PPIP was endorsed as the primary data collection tool for the tri-annual report for the National Perinatal and Neonatal Morbidity and Mortality Committee (NaPeMMCo) established in 2008. NDOH further endorsed PPIP and Child PIP in 2012 and 2017, respectively. The Strategic Plan for Maternal, Newborn, Child and Women’s Health (MNCWH) and Nutrition in South Africa 2012–2016 additionally sought to institutionalize reviews of maternal, perinatal, neonatal and child deaths – thus making PPIP and Child PIP mandatory.

To further support implementation, several additional national-level committees have been established. For PPIP, a national level task team, NaPeMMCo has promoted the scaling up of the programme with the view to having as many facilities as possible submit complete data. For Child PIP, an Executive Committee and Technical Task Team were established to ensure that solutions are updated, training provided, and national government advised on programmatic findings and developments. At a Provincial level, the provincial government and District Clinical Specialist Teams provide support to and quality control of implementation. This includes national-level mortality meetings that, as described above, have encouraged engagement and motivation for the solutions’ ongoing use. While sub-district and facility-level meetings could continue without external funding, respondents spoke about the value of the national meetings that may not be sustained in the absence of external funds.

At the facility level, the implementation of PPIP and Child PIP reporting systems occurs vertically, in a broader organisational context where vital events are also captured in the DHIS2 as noted above. In most cases, facility-level healthcare workers support day-to-day implementation of PPIP and Child PIP, with administrative and managerial staff assisting with some of the data capture. Some provinces have employed full-time Child PIP Coordinators to coordinate collection and collation of data across all facilities; a role seen as highly valuable to the success of the programme, as has been the case in Mpumalanga (504_ Govt. implementor).

Despite their perceived benefits, respondents spoke about facility-level barriers to implementation. Chief among these is the person-time to enter programme data, which is particularly challenging in the South African public health system where human resources and facility providers are chronically overburdened by existing clinical and administrative demands. This is particularly true for Child PIP, which relies more heavily on doctors rather than other cadres of health workers to assess the modifiable factors that led to child mortality. As one Child PIP implementer explained, “ … at individual site level where if you have one community service doctor looking after a 30 bedded paediatric ward with desperately sick children every day of their lives, it’s not that easy to run an audit - a mortality audit at the same time. So you know - or one - people can just be overwhelmed by the clinical work” (505_ Govt. implementor).

### MomConnect

#### Value proposition

MomConnect was buoyed by support from the highest levels of the health department. The Minister was described as thinking MomConnect “was a good tool to reach pregnant women”, one that he “wanted,” and sought to “protect” even in the face of significant budget constraints (101_Govt. NDOH, policy). His interest in the solution was underpinned by South Africa’s poor performance in attaining Millennium Development Goals 4, 5, and 6. The specific components of MomConnect—maternal Short Message System (SMS) messaging, Helpdesk, pregnancy registration – were established in response to the Minister’s desire to develop a platform to connect to and collect feedback from pregnant and postpartum women. From a donor perspective, MomConnect was spoken about as an attractive funding opportunity because of its perceived value to NDOH. One of the long-standing donors of MomConnect explained: “I mean the dream of most donor funded projects is that they will eventually become government owned projects” (201_ donor).

Despite enthusiastic support from donors and the highest levels of the health department, concerns were voiced about the lack of evidence underpinning its endorsement, some saying that the decision “wasn’t evidence based. It was gut feel” (101_ Govt. NDOH policy). The same participant described the challenge in balancing the funders’ interest, political imperative and generating “good evidence”- where in this case the generation of evidence linking exposure to health information messages to key health outcomes was deemed too slow. There have been more recent efforts to address this through implementation research, whose findings were used to support learning in an organic process of course correction as the programme unfolded (102_Govt.NDOH policy). In relation to engaging women themselves, a senior NDOH policy maker confessed: “One thing we didn’t do — which in retrospect we should have done … is that we should have asked the users what they wanted” (102_Govt. NDOH policy). Although there was no user testing in the design of the programme, a senior policy maker compared MomConnect to the Coca Cola brand in terms of its extensive national visibility and generally positive public perception (101_Govt. NDOH, policy).

The initial push to scale MomConnect in the absence of evidence on value for money or impact linking messaging exposure to changes in health outcomes has been a long-term impediment to sustainability. Evidence on costs and cost-effectiveness are seen as integral to the investment case required by Treasury to support sustained government investment. Understanding the value, or impact, of each dollar spent is seen as important to supporting ongoing buy-in as well from non-government stakeholders, especially given the expense of running MomConnect at national scale (404_tech. company; 202_ donor).

#### Actors

As mentioned above, respondents pointed to the then Minister's commitment to MomConnect as the most significant driver of the decision to scale the programme nationally. This engagement began with the Minister’s exposure to a small scale SMS programme being piloted in South Africa, that was followed by his request to senior leadership in the NDOH at the Deputy Director level to initiate a similar but bespoke initiative. Following a commissioned review of maternal messaging programmes, and through an iterative process of engaging with key stakeholders including technology partners, donors, UN agencies, and program implementers, MomConnect was born (401_NGO tech. assistance).

The Minister’s continued engagement and vocal support in the launch and implementation of MomConnect was seen as vital to the programme’s successful roll-out and scale-up (403_tech. partner). Further senior level support in the NDOH included a Deputy Director-General (DDG) who seconded a full-time senior technical advisor to help to establish and run the MomConnect Task Team – a consortium of technology, programme, donor, and academic partners. Continuity in these two positions – DDG and Technical Advisor—have been integral to the programme’s implementation and continuity, particularly in the face of changes in the Minister of Health leadership and fluctuations in funding. The DDG continues to track MomConnect closely through receiving and reviewing weekly emails detailing registration and deregistration numbers and HelpDesk compliments and complaints (102_ Govt. NDOH policy).

Outside of NDOH, MomConnect’s programmatic success is attributed to a strong consortium of local technology partners and external donors. As part of the former, respondents point to a trio of local partners each with complementary experience and expertise. This included one organization with prior experience implementing a maternal messaging program at scale, who handled the ‘front-end’ of the program including registrations and message delivery. A second technology partner was the chief architect behind the system design and architecture of the MomConnect technical platform, while a third partner handled technical interoperability with DHIS2 data on antenatal care attendance in the public sector.

The scale-up of MomConnect is closely tied to the availability of external funding. To date, this includes contributions from a consortium of local and international funders, including the United States PEPFAR, the United States Agency for International Development (USAID), the Johnson & Johnson Foundation, UNICEF South Africa, ICF International, Elma Philanthropies, Cardno Emerging Markets and Regenstrief Institute.

Close ties *between* donors was reported to promote continuity in funding to the programme as well as improved accountability. Donors spoke about the value of coming together to share in the risk of funding a programme, but also in attempt to reduce fragmentation in the digital health space by trying to “really make a *few* things fly” instead of donors “going rogue” through funding multiple different programmes (201_donor).

MomConnect registration and messaging data depend on mobile network operators (MNOs)—Cell C, MTN, Telkom Mobile and Vodacom in South Africa. Government stakeholders noted that initial engagement with MNOs had been challenging, with “the reality [that] you can’t get in the door [to engage MNOs] even if you are the NDOH and there’s been ministerial letters from the Minister of Health to the Minister of Post and Telecommunications to try and deal with this issue [of high costs]” (SA_101, NDOH policy). A donor also spoke about how when they initially began funding MomConnect, they actively engaged with MNOs to negotiate cost reductions in sending SMS’s; a task in which they eventually succeeded (202_donor).

#### Technology

A key strength of the MomConnect technology was that it was designed to ensure that users with any type of mobile phone can use the service. However, the decision to use Unstructured Supplementary Service Data (USSD) presented some registration challenges. Frequent time-outs coupled with the failure to follow-up on attempted or incomplete registrations mean that an estimated 25% of attempted registrations fail – a factor which impedes program coverage (405_tech.company). While core data elements are routinely synthesized as part of a dashboard, challenges with accessing beneficiary-level data on registrations and message delivery persist; limiting efforts to monitor data flow, including registration failures. Further challenges with data governance were also noted by respondents. Data storage is currently fragmented, and overarching consent procedures need to be strengthened to ensure compliance with the Protection of Personal Information Act 4 of 2013 (POPIA).

The costs associated with running MomConnect represent an ongoing challenge as the cost assigned by MNOs for sending out SMS messages remains high. From one funder’s perspective: “Cost reduction is critical and corporate partnerships I think are critical. Like I said if you could have a partnership where inventory costs are reduced for example or hosting is paid for or all of these sorts of things they dramatically reduce the cost of the program and make it more sustainable” (404_tech. company). There have also been attempts to explore alternative delivery channels to SMS to reduce costs, including WhatsApp but these have been implemented with varied success (101 _Govt. NDOH Policy; 102_ Govt. NDOH policy).

#### Organisational context

The most notable contextual factor that has enabled the successful scale-up of MomConnect is national-level governmental leadership, including that of the Minister and MomConnect Task Team that has facilitated implementation and coordination amongst partners and advised the government. There is also a strong foundation of policies, including the South African National Health Normative Standards Framework, that has encouraged MomConnect to adopt critical design features that help to enable scale. On the ground, MomConnect’s implementation is occurring in an environment where women have access to mobile phones and high levels literacy which is essential for the successful uptake of SMS content. Nearly all pregnant women attend antenatal care (ANC) in the public sector during pregnancy, which allowed for a face-to-face encounter where healthcare workers could promote MomConnect to these beneficiaries, seek consent, and facilitate registration to the programme.

Despite these enabling factors, several factors may continue to challenge MomConnect’s ongoing implementation, further expansion and sustainability. Implementation is occurring as part of routine ANC in government health facilities that receive high patient volumes and are understaffed. Time to introduce and register women to MomConnect invariably competes with time allotted for service provision, including counselling, screening, and pregnancy monitoring. Registration was originally intended to be done by a nurse with the woman on her first antenatal visit using the woman’s personal mobile phone, but as one respondent described it, “that was very, very rare”. Instead, the majority of registrations occur in a “batch”, with individual respondent details recorded first on paper and later entered *en masse* by a facility staff member using their own or a facility mobile device. According to respondents, this process increases the potential for registration errors but also has implications for POPIA which requires that consent be recorded as deriving from the respondent’s personal mobile device – not that of a third party. Efforts to have women complete their own registration presented additional challenges as women often could not understand the English prompts or did not manage to input all of their information before USSD sessions timed out (405_tech. company).

### CommCare

Unlike the cases presented above, CommCare was selected rather than implemented at scale, and at the time of interviews, was yet to be deployed. We therefore focus on understanding the processes underpinning decisions to use CommCare as a solution for CHWs at national scale.

#### Value proposition

South Africa receives substantial funding from foreign donors for HIV prevention and treatment including antiretroviral therapy (ART). Highly variable quality of HIV services and poor adherence to ART, coupled with gaps in performance monitoring and reporting, have driven donor interest in digital solutions for data capture, particularly among CHWs. A funder described the value of digitising what had been paper-based reporting: supporting the provision of high quality care through helping to more efficiently organise patient data, but also allow for easier monitoring and evaluation of patients (203_ funder).

Following the release of the 2017 WBPHCOT Framework and Strategy, the NDOH, with donor support, commissioned a landscape review of existing digital solutions for health workers in South Africa to produce a short list of the top digital solutions based on pre-specified criteria [19]. NDOH wanted to implement an electronic solution to get data from the “army of community health workers” (203_ funder) deployed to address health issues in South African communities, as well as the data that they collect and trace on their patients (401_NGO tech. assistance).

CommCare was reported to have limited prior implementation history in South Africa, particularly when compared to locally grown alternatives such as Aita.Health and Catch & Match described in the Digital Square Report [19]. CommCare’s selection for implementation in South Africa was driven by its demonstrated use for CHW data capture and decision-support at scale in other PEPFAR supported countries in the region (203_ funder). The ability to draw from experiences elsewhere and demonstrate the effective capture of similar data elements needed for routine reporting was seen as integral for its selection. The further ability to rapidly mobilise its deployment in South Africa and the narrow timeline available for implementation was also reported to be a significant factor further underpinning its selection (301_tech. partner; 303_tech. partner; 306_tech. partner).

CommCare was signed off by the NDOH who hoped that 57,000 CHWs would use the solution by early 2020, first in 27 pilot districts, but later in all 52 (102_Govt. NDOH, policy). The NDOH initially requested that CommCare be configured to support the data capture needs of the full scope of CHW-led service delivery. While NDOH was interested in the development of an HIV specific data capture digital solution, their preference was for the implementation of a digital platform for data capture inclusive of other conditions monitored by CHWs (SA_301, tech partner). This potential misalignment between the priorities of different actors added a layer of complexity and ambivalence to the process. As a representative from the Centers for Disease Control and Prevention (CDC) put it:*“*PEPFAR’s funding is for HIV, so we’ve had to do this dance of like how do we uhm support the government’s position, but also be able to report back to the American congress over how HIV funds are being expensed” (301_tech. partner).

Additional concerns were flagged about the contextual fit, evidence for and process of selection. A senior academic with extensive experience in digital health highlighted the limitations of doing such assessments solely based on prespecified, technical metrics, rather than taking context and “localisation” into account (302, Govt. Technical assistance). Another respondent explained: “I saw the evaluation [digital square report]. That was the last I heard … and I heard they were talking about a scale of about 7000 [CHWs]. And this is - this is part of my criticism for the whole process because where is the pilot? Where is the learning? Where are the evidence-based comparisons? CommCare hasn’t been used at any scale in the province as far as I know of. Where was the consultation?” (SA_307, govt. technical assistance). As one technical partner explained, if a programme is to be sustainable it should be driven by what NDOH “believe something can make a real difference. Because that’s ultimately gonna drive the process and then the app is the very last thing that comes, you know? Because that can be filled in in many different ways” (403_tech. partner).

#### Actors

Two primary actors drove decisions to scale-up CommCare: NDOH and donors. Beyond selection, the NDOH has played a limited role in supporting implementation (301_tech. partner). An implementing NGO has worked closely with the technical partner who developed CommCare to tailor its use in the South African context. PEPFAR, the primary donor behind this use of CommCare in South Africa, drew on their pre-existing relationship with the implementing NGO, having worked together on other projects in South Africa before. The implementation partner and technical partner bring a wealth of experience working in South Africa, particularly in HIV and TB. In their view, they have the “local knowledge” to carry out successful implementation (301_tech. partner; 303_tech. partner).

Similarly, the technology partner’s past experience working on similar programmes globally at scale, with donors, governments, and NGOs, was self-reported as a positive attribute. Engagement, and importantly “managing the expectations” of a range of partners, plays a significant role in the implementation, roll-out and on-going functionality of a programme—from talking to representatives from the NDOH, to ensuring the technical capacity of employees, to engaging and training CHWs themselves (303_tech. partner).

While there had been extensive communication with government, technology and implementation NGOs about CommCare, one respondent explained that CHWs themselves had not yet been consulted: “what’s been challenging on this project is we’ve had very little exposure to what’s happening on the ground … we haven’t had the chance to for example speak with CHWs directly or frontline workers yet” (SA_304, tech partner). Respondents spoke about this as one of the challenges widening the gap between national-level policy and planning, and the implementation on the ground (301_tech. partner; 304_tech. partner; 306_tech. partner).

#### Technology

CommCare is a data capture and decision-support application (app) with a flexible design structure that can be modified based on guidance from donor and implementing partners (301_tech. partner; 303_tech. partner; 306_tech. partner). For basic use, users pay a monthly or annual fee and can modify the app directly, obtain access to data on pre-scheduled exports, integrate with excel dashboards, and generate workforce monitoring reports. For more complex needs, the fee structure is higher as the technology company plays a more direct role in modifying the app to fit workflow and data capture needs, including implementation support and monitoring and evaluation (SA_301, tech partner). In close consultation with NDOH and donors, technical and implementing partners worked to tailor the app for CHWs and HIV service delivery activities; support training, data extraction and visualisation; and ensure adherence to donor data reporting requirements.

CommCare is not interoperable with routine health information systems in South Africa including DHIS2, and in contrast to MomConnect, is not adherent to the South African National Health Normative Standards Framework (NHNSF). Once collected, data captured through CommCare are housed in the technology company’s cloud that is based in the United States of America. While respondents noted that ideally data should all be “locally owned”, in other words housed in South Africa, ideally within the NDOH, such a data repository does not currently exist. With POPIA set to take effect in 2021, broader systems for data governance including consent, capture storage and access to personal data will need to be reassessed (301_tech. partner; 303_tech. partner).

#### Organisational context

CommCare’s implementation in South Africa is occurring while health systems support for CHWs, including timely and adequate remuneration, transportation, supervision, and adequate drugs and supplies, remain outstanding. Further limitations in CHW digital literacy, coupled with the broader support requirements needed to effectively govern technology use in government services were raised by respondents as potential impediments to sustained use (308_academia; 309_tech.partner). An academic with extensive experience working with CHW programmes in South Africa spoke about how if 57,000 tablets are distributed to individual CHWs, within a few weeks several thousand will be “lost, stolen, gone” and another few thousand will break, limiting the number of functional tablets “out there” (601_academia).

In addition to health systems limitations constraining scale up of CHWs, CommCare remains one of several apps used by CHWs for data capture. Alternative data capture and decision-support tools developed by local technology and implementing partners remain in use throughout the country. The implications of the use of CommCare in contexts where alternatives remain in use is unknown. One respondent clearly highlighted the confusion that this could create for health managers and CHWs on the ground: “Where’s the coordination there? If I was a provincial HOD (Head of Department), I wouldn’t know what to make of all of this. Do I now use CommCare for community health workers, but for all TB I use Catch & Match? Why do I use CommCare if I can use Aita.Health because it is being used in Gauteng?” (307_govt. tech. assistance).

While CommCare has been signed off by the NDOH, it has not been provided the same degree of support and oversight observed in the case of MomConnect. In other countries, governments insisted on parameters for promoting CommCare’s sustainability from the outset, by training government employees on how to use and amend the CommCare backend without assistance from the technical partner (SA_301, tech partner). This is not the case in South Africa, where donors and technical partners reported that there does not appear to be a clear sustainability plan. As one funder put it: “We’ll be supporting it [CommCare] through the next - through the current year. Through [the implementing NGO], but we don’t have any idea of funding from beyond next September. So we’re not really able to - you know maybe NDOH has a discussion or arrangement with CommCare directly, but we haven’t - as far as I know - we haven’t been told of that.” (203_ funder).

## Discussion

We presented our findings using a conceptual framework that highlights four domains (value proposition, actors, technology and context) to explore how and why selected cases of digital health solutions have scaled in South Africa. The development, implementation and continued use of PPIP and Child PIP was led by clinicians, who saw the need to digitise mortality data to assist in clinical auditing processes [20, 21]. The scaling of MomConnect, on the other hand, was primarily facilitated by top-down political will, including the then-Minister of Health, whose support allowed for the establishment of a government-led MomConnect Task Team and encouraged funders’ continued investment. CommCare was selected by NDOH, informed by donor preferences, as the platform to digitise HIV and TB information collected by CHWs in South Africa.

Across cases, the value proposition behind the scaling of digital health solutions is inextricably linked to the views and input of key actors in various leadership positions, including clinicians, donors and NDOH leaders and representatives, who have the power to spearhead the implementation and continued use of particular digital solutions. The meaning of ‘value’ was also slightly different in each case. This is in part linked to a digital solutions’ impact on health- something for which there remains too little evidence [8]. PPIP and Child PIP are the oldest solutions with the high levels of buy-in from health workers who could tangibly see the solutions’ value in their function and utility in making clinical audits and reporting simpler and more accessible to a broader audience. For MomConnect and Commcare, the decision to scale these solutions was more closely linked to high-level political buy-in and decision-making, as well as donor preferences and capacity to fund particular solutions over time. In each of these cases, shifts in leadership, competing priorities at NDOH as well as the generally dynamic nature of the use and relationships between actors over time highlight “implementation as trajectories of problems and solutions” [12]. Here, the decision to implement a digital solution may not anticipate all the adjustment and problems that must be collectively resolved once implementation starts.

For a solution to achieve scale and sustain use, the technology it uses need not be complex, but does require continual attention and resolution. Importantly, the technology needs to be integrated into existing programmes and related workflows (ie. not limited to data systems), without adding further complexity to the systems in which they function [22]. PPIP and Child PIP are relatively technologically ‘simple’ compared to the CommCare platform, which could be seen as the most developed and versatile technology. Due to high mobile phone penetration, MomConnect reaches a significant proportion of women in South Africa, but high costs and technological challenges remain [2, 3]. Challenges with data governance also raise concerns across cases, even in the case of MomConnect that has some alignment to legislation [23]. Moreover, interoperability endures as an unaddressed issue critical for sustainability [4].

The organisational context of the South African health system has significant potential to threaten the sustainability of all digital solutions operating within it. While digital solutions might help to address certain technical problems, they cannot eliminate health systems challenges [12, 24], nor, crucially, should they be expected to do so [8]. Given the complexity and uncertainty that characterises the systems in which these digital solutions operate, there is additional pressure for solutions to be especially dynamic and adaptive so as to overcome these hurdles. Of central concern are human resource capacity, as well as funding and service delivery processes required to deliver care [25]. Here there is a significant and persistent tension between shorter-term, donor-driven decision-making about what gets scaled based on their resources, and government grappling with what and how to invest to sustain particular digital health solutions over the long -term. Without adequate financial and human resource capacity, the government is too often in a position of reliance on donor support to ensure that health services are adequately provided, and is likely to continue to struggle with how and when to take steps towards replacing donor contributions [26, 27].

Our findings highlight that there is no single pathway for digital solutions to achieve scale or sustainability, but instead point to the ways that a series of factors may converge to support or hinder this outcome. Given that health systems are complex non-linear systems, digital solutions are more likely to achieve scale when the technology does not add further complexity to the system, the value proposition is clear and likely to sustain investment, and where the solutions do not require innovation that stretches the existing system too far [10, 15]. In line with Greenhalgh (2018) and Spicer et al. (2014), our findings point to the value of engaging in the “relationship work” of collaboration with all stakeholders, including technical partners, implementers and end users is also critical to the process of achieving scale and sustainability [28]. Additionally, these issues cannot be adequately addressed without conscious investment in robust monitoring and evaluation of each of the digital solutions concerned, especially with respect to their impact on health [29, 30].

In addressing the broad question of scale-up of digital interventions, more research is needed exploring the interface between technical questions of design, delivery and system innovation and maintenance on the one hand, and interpersonal and political questions of who becomes influential and how in relation to facilitating or hindering scale-up and sustainability, on the other. These questions also warrant exploration in the context of potential shocks to the health system, including those recently experienced in relation to the COVID-19 pandemic. Future research should continue to explore the ongoing evolution of the development and use of digital health solutions, including with . end users given that they determine final implementation.

There are several strengths and limitations to this research. The presentation of varied cases of digital solutions allows for productive cross-case comparison. However, some of the cases have a history that predates their digital nature and therefore are not usually identified as purely digital health solutions. A further limitation is that we did not specifically seek to include end users in our participant sample. Their perspectives could have added to our understanding of the ‘value proposition’ behind each digital solution. Some of the interviewees could be understood as advocates of programmes they spoke to which could have led to a biased view, although we did seek perspectives from a range of stakeholders. Co-authors have experience evaluating some of the digital solutions examined, and therefore were able to place what respondents said at face value in broader context. Nonetheless, the study is still retrospective in nature, relying on respondent recall, rather than a prospective tracking of decisions and experiences as they unfolded.

## Conclusions

Our findings highlight that there is no single pathway to achieving scale up or sustainability, and there will be successes and challenges regardless of the configuration of the domains of value proposition, technology, actors and organisational context. While scaling and sustaining digital solutions can be technologically complex, perhaps more complex are the idiosyncratic factors and nature of the relationships between actors involved. These actors, whether individuals or collections of individuals, have multiple motivations, values and views that may be difficult to balance. These relationships are also shaped by the interplay of different forms of power at multiple levels, which all warrant consideration. Scaling up and sustaining digital solutions need to account for the interplay of the various technical and social dimensions involved in supporting digital solutions to succeed, particularly in health systems that are themselves social and political dynamic systems.

## Data Availability

The datasets used and/or analysed during the current study are available from the corresponding author upon reasonable request.

## Appendix

**Table 1:** Summary description of four case studies of digital solutions in South Africa

Case study	Why? Value proposition	What? Components/ Functions	Who? Actors	When? Timeline of key milestones	Where? Geography and Coverage
***Perinatal Problem Identification Programme (PPIP)***	PPIP was developed to address the data needs of clinicians to understand the reasons for perinatal deaths to inform further action	An electronic perinatal mortality auditing tool: Captures and allows for analysis of perinatal (22 weeks’ gestation- 7 days after birth) and maternal deaths, as well as other related morbidity information. PPIP focuses on: 1) Numbers of deliveries, stillbirths and early and late neonatal and maternal deaths (“Identification”) 2) Causes of death 3) Avoidable factors (carer, health care personnel or health system). The data is used during audit meetings to identify solutions.	Adopters: facility health providers who are often nurses supported by doctors. Tech: Clarion and later Windows. Implementation: National Department of Health (NDOH), Provincial Department of Health (DOH) External Funders: Limited overall funding, some South Africa Medical Research Council (SAMRC) and Centre for Disease Control (CDC) funding.	Pre-1994- paper-based recording of ‘avoidable’ factors 1994- first digital version of PPIP developed [20] 1995–2000- scale-up driven by champions; scale increases with time 2003- Saving Babies Task Team established 2008- National Perinatal and Neonatal Morbidity and Mortality Committee (NaPeMMCo) established 2012- PPIP mandatory for all public health facilities where deliveries and care for new-borns occurs [31]	Nationally endorsed but unevenly used across the country. In 2015, it included 588 participating sites, covering 75.6% of government facility births as per District Health Information System (DHIS) [31]. Most complete PPIP data: Western Cape, Mpumalanga, North West and Free State provinces. Least complete PPIP data: Eastern Cape, Gauteng and KwaZulu Natal provinces.
***Child Healthcare Problem Identification Programme (Child PIP)***	Child PIP was implemented to improve the quality of care that children were receiving in public hospitals, based on lessons learned from a review of deaths in these institutions	An electronic child mortality auditing tool designed to assist health providers better understand and improve care for infants and children from birth to 18 years Child PIP focuses on: 1) Numbers of child deaths 2) Causes of death 3) Modifiable factors (carer, health care personnel or health system).	Adopters: more clinician driven than PPIP Tech: Jembi Implementation: DOH Funders: limited CDC, GlaxoSmithKline (GSK) funding	1994- first digital version of PPIP developed [20] Early 2000s- Dr. Angelica Krug piloted in North West province [32] 2004- Child PIP field testing led to first Saving Babies Report	Nationally endorsed but unevenly used across the country. Approximately 80% coverage of hospitals across the country. Rapid scale-up from 4 pilot sites in 2002 to over 50 in 2008.
***MomConnect***	MomConnect was primarily developed to address the failure to meet the Millennium Development Goal targets related to maternal and child survival by 2012, as part of a suite of interventions [5]	A pregnancy registration and mobile messaging service for pregnant and postpartum women in the public sector. Messages to women (a) provide stage-based health information messages bi-weekly and (b) a helpdesk to enable users to ask questions, and provide compliments or complaints about services received	Adopters: Health workers who register women; pregnant women who receive messages and send feedback Tech: Jembi, Praekelt Implementation: NDOH, DOH, Praekelt Funders: USAID/ PEPFAR; Johnson&Johnson; Elma Philanthropies; UNICEF; NDOH Other: Mobile Network Operators	2013- MomConnect Taskteam established 2014- MomConnect officially launched in August	Nationwide, includes over 2 million pregnant women attending first antenatal appointment (but only 60% of all pregnant women), provided by 30,000 nurses, over 4000 public health facilities across 52 districts [5].
***CommCare***	CommCare was originally developed to assist frontline health workers deliver services to populations in low-resource settings	A digital application for CHWs that includes primarily features for data capture and secondarily for decision support, in this case for HIV and TB	Adopters: CHWs Tech: Dimagi, Jembi Implementation: Aurum Institute supported by NDOH Funders: PEPFAR, UNAIDS	2007- CommCare tool launched by Dimagi. Inc. [33] 2008- CommCare tested in two AIDS treatment sites in SA [34] 2018- Digital Square Report describing significant digital solutions in SA [19]	At the time of interviews, implementation was not initiated. It was slated for late 2019 and projected to scale-up to 10,000 users over a nine-month period, starting in 12 out of 27 health districts in Gauteng and KwaZulu Natal provinces (SA_301_CommCare, tech partner).

## Abbreviations

ANC: Antenatal Care
ART: Antiretroviral Therapy
CDC: Centers for Disease Control and Prevention
Child PIP: Child Healthcare Problem Identification Programme
CHW: Community Health Worker
DDG: Deputy Director-General
DHIS2: District Health Information Software version 2
MNCWH: Maternal, Newborn, Child and Women’s Health
MNOs: Mobile Network Operators
NASSS: Greenhalgh’s Non-Adoption, Abandonment, Scale-Up, Spread, And Sustainability Framework of technology scale-Up
NDOH: National Department of Health
NHNSF: National Health Normative Standards Framework
NaPeMMCo: National Perinatal and Neonatal Morbidity and Mortality Committee
NGOs: Non-governmental Organisations
PPIP: Perinatal Problem Identification Programme
PEPFAR: President’s Emergency Plan for AIDS Relief
POPIA: Protection of Personal Information Act 4 of 2013
SMS: Short Message System
SAMRC: South African Medical Research Council
USAID: United States Agency for International Development
UCT: University of Cape Town, South Africa
USSD: Unstructured Supplementary Service Data
WBPHCOTs: Ward-Based Primary Healthcare Outreach Teams
WHO: World Health Organization
